# Acute and chronic rhinosinusitis and allergic rhinitis in relation to comorbidity, ethnicity and environment

**DOI:** 10.1371/journal.pone.0192330

**Published:** 2018-02-05

**Authors:** Ruth Hoffmans, Alex Wagemakers, Cornelis van Drunen, Peter Hellings, Wytske Fokkens

**Affiliations:** 1 Academic Medical Centre, Amsterdam, The Netherlands; 2 UZ Leuven, Leuven, Belgium; Tongji Hospital of Tongji Medical College of Huazhong University of Science and Technology, CHINA

## Abstract

**Background:**

This study was conducted to assess the effect of comorbidity, ethnicity, occupation, smoking and place of residence on allergic rhinitis (AR), acute rhinosinusitis (ARS) and chronic rhinosinusitis (CRS).

**Methods:**

A GA^2^LEN (The Global Allergy and Asthma European Network) screening questionnaire was sent to a random sample of the Dutch population (n = 16700) in three different areas of the Netherlands.

**Results:**

Fifty percent (8347) of the questionnaires sent were returned. A total of 29% respondents (27–31% in different areas) met the criteria for AR, 18% (17–21%) for ARS and 16% (13–18%) for CRS. Risk factors for AR were itchy rash, eczema, adverse response after taking a painkiller, asthma, CRS and ARS. Moreover, the risk of AR was twice as low for full-time housewives/househusbands than for people with jobs. The risk of ARS or CRS was significantly higher in respondents with a doctor’s diagnosis of CRS, AR, itchy rash or smoking. The risk of CRS was also significantly higher in respondents with an adverse response after taking painkillers, active smoking or asthma. Caucasians are generally less likely to have AR or CRS than Latin-Americans, Hindustani and African-Creoles, and more likely to have ARS than Asian, Hindustani, Mediterranean and African-Creoles.

**Conclusions:**

This study found shared and distinct risk factors for AR, ARS and CRS and therefore provides support for the belief that they have shared symptoms but are different diseases with different aetiologies.

## Introduction

Allergic rhinitis (AR), acute rhinosinusitis (ARS) and chronic rhinosinusitis (CRS) are common upper airway diseases. [1–4] According to the European position paper on rhinosinusitis and nasal polyps (EPOS), rhinosinusitis is clinically defined as inflammation of the nose and the paranasal sinuses characterised by two or more symptoms, one of which should be either nasal blockage/obstruction/congestion or nasal discharge (anterior/posterior nasal drip) and/or facial pain/pressure and reduction or loss of smell, combined with objective signs of disease identified by endoscope or CT scan. The definition without objective signs is used in epidemiological studies. When the onset of these symptoms is acute and when they are present for less than twelve weeks, the diagnosis is ARS. When they persist for more than twelve weeks, the diagnosis is CRS. [2]

AR is clinically defined as a symptomatic disorder of the nose induced after allergen exposure by an IgE-mediated inflammation of the nasal membranes. The symptoms include rhinorrhoea (anterior or posterior), nasal congestion, nasal itching, and sneezing. [5] There is no uniform definition for epidemiological studies. Different definitions have been used in questionnaires in previous studies. [5, 6]

There is a lot of data about the effect of comorbidity (eczema, urticaria and asthma, for example), ethnicity, occupation, smoking and place of residence on the incidence of AR [5], but less is known about the effect of these factors on CRS [2] and little is known about ARS.

The GA^2^LEN survey was conducted under the auspices of The Global Allergy and Asthma European Network (GA^2^LEN). The associated questionnaire was designed to focus specifically on upper airway symptoms and particularly upper airway disease like rhinitis and rhinosinusitis, but also on some gaps in our scientific understanding of allergic disease and some risk factors such as adverse response to painkillers, occupation, ethnicity, smoking exposure, age and gender.

There are theories about the association between AR and ARS and CRS. One theory is that allergy causes swelling of the mucosa, which obstructs the ostium of the sinuses and impairs mucocilliary transport, and possibly induces rhinosinusitis. [2] Another theory argues that there is significantly more inflammation (eosinophils) in the maxillary sinus of allergic patients during the season than out of season. [7, 8] Pathophysiological processes that involve the upper airway generally affect lower airway disease. Mucosa in the ear, nose, sinus and lower airways is often inflamed at the same time. The majority of patients with asthma also have allergic rhinitis. Support for the unified airway theory is found in epidemiological studies, in shared pathophysiological mechanisms, and in interactive treatment effects. [5, 9–11]

We wanted to look at whether different areas (with different levels of air pollution) in the Netherlands (Amsterdam and the east of the Netherlands) and/or ethnicity could play a role in the prevalence and severity of ARS, CRS and AR. This study was conducted to assess the relationships between AR, ARS and CRS and comorbidity, ethnicity, occupation, smoking and place of residence.

## Methods

### Study design

**Fig 1:**
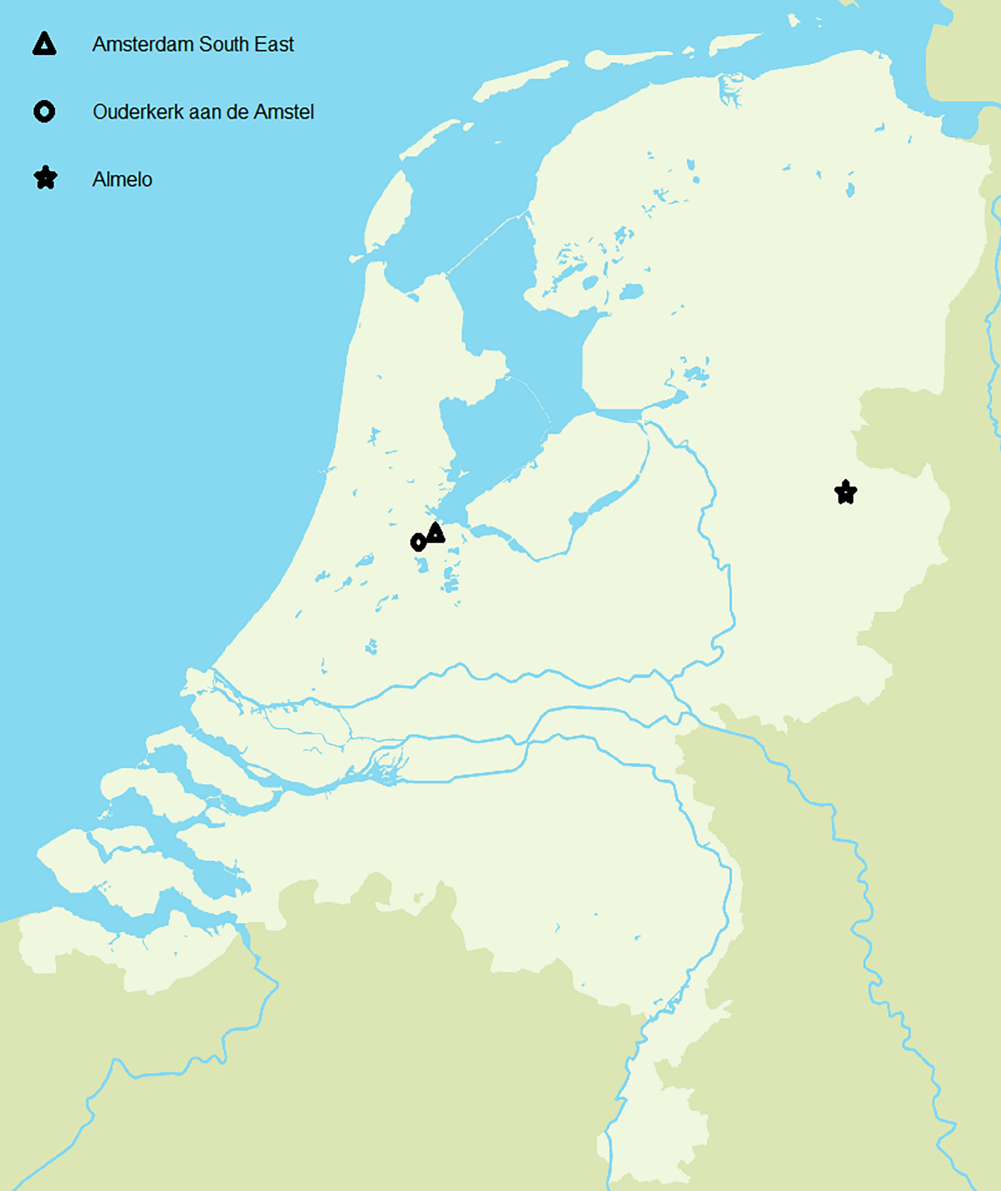
Map of the Netherlands with the three different areas.

Most of the data for the present study were obtained using the GA^2^LEN questionnaire, which consists of 22 questions. The questionnaire was sent to a random sample of the Dutch population in three different areas of the Netherlands (Fig 1) with different geographic locations, housing, population densities, ethnic profiles:

1. Ouderkerk aan de Amstel, a suburban village close to Amsterdam (545 inhabitants/km^2^): 5000 questionnaires
2. Amsterdam South East (urban area with many different ethnicities, 4704 inhabitants/km^2^): 6700 questionnaires
3. Almelo, a city in a more rural area in the east of the Netherlands (1077 inhabitants/km^2^): 5000 questionnaires

In the surveys in Amsterdam and Almelo (sent in 2009), we included extra questions about acute rhinosinusitis (ARS) and ethnicity alongside the questions about chronic rhinosinusitis (CRS) and allergic rhinitis (AR).

The questionnaire can be found in the supporting files (S1 Appendix). We sent it up to three times if there was no response.

### Relevant definitions based on the questions in the questionnaire

AR: A positive answer to the question: Do you have any nasal allergies including hay fever?

ARS: A positive answer to the question: In the past twelve months, did you have at least one episode of at least ten days with a blocked nose, discoloured nasal discharge and pain or pressure in the sinuses?

CRS: A combination of two positive answers to the next questions (with at least a positive answer to either A or B):

- Has your nose been blocked for more than twelve weeks in the last twelve months?
- Have you had discoloured nasal discharge (snot) or discoloured mucus in the throat for more than twelve weeks in the last twelve months?
- Have you had pain or pressure around the forehead, nose or eyes for more than twelve weeks in the last twelve months?
- Has your sense of smell been reduced or absent for more than twelve weeks in the last twelve months?

A doctor’s diagnosis of CRS: A positive answer to the question: Has a doctor ever told you that you have chronic sinusitis?

Itchy rash: A positive answer to the question: Have you ever had an itchy rash that came and went for at least six months?

Eczema: A positive answer to the question: Have you ever had eczema or any kind of skin allergy?

Adverse response after taking painkillers: A positive answer to the question: Have you ever had any difficulty with your breathing within three hours after taking a painkiller?

Active smoking: A positive answer to the questions: Have you ever smoked for as long as a year? AND Have you smoked at all in the last month?

Asthma: A positive answer to the question: Have you ever had asthma? AND one of the following:

- Have you had wheezing or whistling in your chest at any time in the last twelve months?
- Have you woken up with a feeling of tightness in your chest at any time in the last twelve months?
- Have you been woken by an attack of shortness of breath at any time in the last twelve months?
- Have you been woken by an attack of coughing at any time in the last twelve months?

### Ethics statement

Our institutional review board (ethics committee) decided that their approval was not needed to start this study because participants were not subject to any intervention.

### Statistical analysis

Statistical analyses were conducted with SPSS 21.0 statistical software.

Univariate statistical analyses for all the different variables in each area were completed. The percentages were calculated using the frequencies and total available data for each area and variable (without missing values). Significant differences between the areas were calculated using Chi-square or ANOVA (Analysis of variance) for each variable.

Univariate analysis was then conducted for the three outcome variables ARS, CRS and AR using Pearson chi-square or t-test depending on binary or continuous data for each variable. The independent variables with a p-value of less than 0.20 in the univariate analysis were selected for multivariate analysis. Multivariate logistic regressions were fitted by using the backward elimination technique based on likelihood ratio to identify factors that affect ARS, CRS and AR separately. The association between independent variables was assessed using odds ratio (OR) and a 95% confidence interval (CI). Correlations were considered to be significant if the p-value was less than 0.05.

## Results

Fifty percent (8347) of the 16700 questionnaires sent were returned (Table 1).

The mean age of the respondents was 46 years (range 6–90); 45% were male.

**Table 1:** Respondents.

Area	Sent	Respondents	Percentage
Ouderkerk a/d Amstel	5000	3192	64
Amsterdam SE	6700	2586	39
Almelo	5000	2569	51
Total	16700	8347	50

### Univariate analysis

Table 2 summarises the results of the univariate analysis.

**Table 2:** Summary of results (univariate).

	Amsterdam SE N (%)	Almelo N (%)	Ouderkerk N (%)	Total N (%)	P
Allergic rhinitis	750 (31) ^$^	699 (29)	810 (27)	2259 (29)	0.005
Doctor’s diagnosis CRS	104 (4)	94 (4)	108 (3)	306 (4)	0.366
ARS	368 (17) ^#^^.^^$^	469 (21)	596 (20)	1433 (18)	0.000
Itchy rash	622 (25) ^$^	601 (24) ^&^	612 (20)	1835 (23)	0.000
Eczema	1095 (45)	1108 (45)	1283 (43)	3486 (44)	0.245
Adverse response painkiller	56 (2) ^$^	54 (2) ^&^	36 (1)	146 (2)	0.002
Smoking (1 year)	1164 (47) ^#^^,^^$^	1319 (52) ^&^	1563 (50)	4046 (50)	0.000
Smoking (active)	595 (43) ^$^	630 (44) ^&^	695 (32)	1920 (39)	0.000
Occupation					0.000
*Employed*	1389 (56) ^#^^,^^$^	1283 (52)	1672 (53)	4344 (54)	0.023
*Self-employed*	117 (5) ^#^^,^^$^	153 (6) ^&^	360 (11)	630 (8)	0.000
*Unemployed*	94 (4) ^#^^,^^$^	55 (2) ^&^	40 (1)	189 (2)	0.000
*Not working because of poor health*	128 (5) ^$^	101 (4) ^&^	67 (2)	296 (4)	0.000
*Full time house person*	101 (4) ^#^^,^^$^	207 (8) ^&^	201 (6)	509 (6)	0.000
*Full time student*	215 (9) ^$^	193 (8)	230 (7)	638 (8)	0.276
*Retired*	312 (13) ^#^^,^^$^	393 (16)	481 (15)	1186 (15)	0.001
*Other*	111 (5)	97 (4)	119 (4)	327 (4)	0.495
Ethnicity					0.000
*Caucasian*	1293 (56) ^#^	1799 (86)		3092 (70)	0.000
*Asian*	141 (6)	104 (5)		245 (6)	0.108
*African-Creole*	354 (15) ^#^	12 (1)		366 (8)	0.000
*Latin-American*	59 (3) ^#^	4 (0,2)		63 (1)	0.000
*Hindustani*	206 (9) ^#^	7 (0,3)		213 (5)	0.000
*Mediterranean*	45 (2)	53 (3)		98 (2)	0.182
*Other*	225 (10) ^#^	117 (6)		342 (8)	0.000
Gender (female)	1453 (57) ^#^	1361 (53)	1748 (55)	4562 (55)	0.058
CRS	450 (18) ^$^	420 (17) ^&^	411 (13)	1281 (16)	0.000
Asthma	185 (8) ^$^	208 (9) ^&^	185 (94)	578 (7)	0.000
Age (mean)	45.4 ^$^	46.5 ^&^	47.0		0.000

# significant difference between Amsterdam Southeast and Almelo

$ significant difference between Amsterdam Southeast and Ouderkerk aan de Amstel

& significant difference between Almelo and Ouderkerk aan de Amstel

A total of 2274 respondents met the criteria for ARS, of whom 841 also met the criteria for CRS. Those 841 patients were excluded from the ARS group since they will have answered ‘yes’ to this question given their CRS. The prevalence of ARS is therefore 18% (1433/8170). The prevalence of CRS was 16% (1281/8227). In total, 29% (2259/7804) of the respondents met the criteria for AR.

All variables were compared for the different areas. AR, ARS, itchy rash, adverse response to painkillers, smoking (active and at least one year), occupation, ethnicity, CRS, asthma and age differed significantly between the areas.

### Multivariate analysis

#### ARS

The risk of ARS was significantly higher in respondents with a doctor’s diagnosis of CRS (OR 2.14), AR (OR 1.70), itchy rash (OR 1.28) and eczema (OR 1.33), in female respondents (OR 1.39) or those with a history of smoking for at least one year (OR 1.22). Caucasians have a significantly higher risk of ARS than people of most other ethnicities in our survey. Getting older reduces the risk of ARS by an OR of 0.99 per year. Table 3 shows all variables significantly related to ARS. No significant relation with work/occupation or place of residence was found.

**Table 3:** Variables related to ARS (multivariate).

Variable	p	OR	95% CI-	95% CI
Doctor’s diagnosis CRS	0.01	2.14	1.17	3.91
AR	0.00	1.70	1.38	2.10
Gender (ref: male)	0.00	1.39	1.14	1.69
Eczema	0.01	1.33	1.08	1.65
Itchy rash	0.04	1.28	1.01	1.62
Smoking (1 year)	0.05	1.22	1.00	1.50
Age (per year)	0.05	0.99	0.99	1.00
Ethnicity (ref: Caucasian)	0.00			
*Other*	0.10	0.74	0.52	1.06
*Latin-American*	0.26	0.54	0.18	1.59
*Asian*	0.00	0.45	0.26	0.76
*Hindustani*	0.00	0.40	0.22	0.74
*Mediterranean*	0.04	0.40	0.17	0.95
*African-Creole*	0.00	0.35	0.21	0.59

#### CRS

The risk of CRS is significantly higher in respondents with a doctor’s diagnosis of CRS (OR 6.83), AR (OR 2.87), asthma (OR 2,36), an adverse response after taking painkillers (OR 2.34), itchy rash (OR 1.71), or active smoking (OR 1.45). Caucasians were less likely to meet the criteria for CRS than people with some other ethnicities (African-Creole, Latin-American, Hindustani). CRS is also less likely in older patients. No significant relation was found with work/occupation, place of residence or gender.

Table 4 shows the variables significantly associated with CRS.

**Table 4:** Variables related to CRS (multivariate).

Variable	p	OR	95% CI-	95% CI
Doctor’s diagnosis CRS	0.00	6.83	3.91	11.94
AR	0.00	2.87	2.11	3.81
Adverse response painkiller	0.01	2.34	1.20	4.54
Asthma	0.00	2.36	1.52	3.66
Itchy rash	0.00	1.71	1.26	2.31
Smoking (active)	0.01	1.45	1.08	1.95
Age (per year)	0.02	0.99	0.98	1.00
Ethnicity (ref: Caucasian)	0.00			
*Latin-American*	0.05	3.56	1.01	12.51
*African-Creole*	0.00	2.53	1.52	4.20
*Hindustani*	0.04	2.04	1.04	4.01
*Mediterranean*	0.20	1.77	0.74	4.26
*Asian*	0.10	1.74	0.90	3.37
*Other*	0.35	0.75	0.42	1.36

#### AR

The risk of AR was significantly higher in respondents with an adverse response after taking a painkiller (OR 4.12), asthma (OR 3.24), CRS (OR 2.24) or a doctor’s diagnosis of CRS (OR 2.29), ARS (OR 1.74), eczema (OR 1.60), or itchy rash (OR 1.43). Active smokers were less likely to have AR (OR 0.74). Full-time housewives/househusbands were significantly less likely to have AR than respondents in employment (OR 0.46). Caucasians generally were less likely to have AR than African-Creoles, Latin-Americans and Hindustanis. Once again, the risk of AR declined with increasing age and no significant relation was found with gender or place of residence. Table 5 lists the variables related to AR.

**Table 5:** Variables related to AR (multivariate).

Variable	p	OR	95% CI -	95% CI
Adverse response painkiller	0.00	4.12	1.71	9.93
Asthma	0.00	3.24	1.98	5.31
Doctor’s diagnosis CRS	0.04	2.29	1.04	5.04
CRS	0.00	2.24	1.34	3.73
ARS	0.00	1.74	1.29	2.35
Eczema	0.00	1.60	1.20	2.13
Itchy rash	0.02	1.43	1.05	1.96
Age (per year)	0.00	0.98	0.97	0.99
Smoking (active)	0.03	0.74	0.56	0.97
Ethnicity (ref: Caucasian)	0.01			
*Latin-American*	0.03	5.13	1.16	22.70
*Hindustani*	0.02	2.35	1.15	4.80
*African-Creole*	0.01	1.97	1.14	3.37
*Mediterranean*	0.29	1.60	0.67	3.83
*Other*	0.12	1.45	0.90	2.33
*Asian*	0.73	1.13	0.57	2.23
Occupation (ref: employed)	0.08			
*Unemployed*	0.20	1.58	0.78	3.21
*Retired*	0.66	1.12	0.68	1.83
*Not working because of poor health*	0.82	1.07	0.59	1.97
*Self-employed*	0.71	0.90	0.53	1.55
*Full-time student*	0.10	0.57	0.29	1.12
*Other*	0.14	0.55	0.25	1.22
*Full-time housewife/husband*	0.02	0.46	0.24	0.87

## Discussion

We evaluated the risk factors for AR, ARS and CRS in an epidemiological study looking at three different locations in the Netherlands.

Most studies in the past have asked subjects whether they had ‘sinusitis’ (diagnosed by a doctor), often without distinguishing between ARS and CRS. [12–14] The present study used the GA^2^LEN questionnaire and so we were able to distinguish between ARS and CRS on the basis of symptoms reported by the patients and a possible doctor’s diagnosis of CRS.

A doctor’s diagnosis of CRS and the diagnosis CRS based on symptoms are obviously related (OR 2,29). But not all participants with symptom based CRS have a doctor’s diagnosis. They may be less care seeking or they may have less severe complaints. Also in the Dutch healthcare system, general practitioners are not always aware of the difference between acute and chronic rhinosinusitis.[15, 16] Therefore the participants that did go to their general practitioner did probably only hear a diagnosis of “sinusitis” and not “chronic rhinosinusitis”.

The strength of symptom based diagnosis of CRS is that participants that are not aware of their diagnosis can be found. We realise that we are not always able to distinguish perfectly between the different diseases: persistent AR and CRS, for example, are not always easy to separate on the basis of symptoms alone.[17] However, using the same GA^2^LEN questionnaire, Tomassen et al. found that 62% of the subjects reporting CRS on the basis of symptoms also had objective abnormalities at endoscopy. [18] A Korean study correlated all the different combinations of CRS symptoms with the findings of nasal endoscopy and found that all combinations with a reduction or loss of smell had the highest OR for a positive endoscopy. [19].

The strength of doctor’s diagnosed CRS is that a professional has combined symptoms and objective findings to make a diagnosis. However part of the patients will not visit their doctor and some doctors will not recognize CRS, leading to an underestimation of the prevalence of CRS.

We have to keep in mind that there may be a participation bias. Individuals with nasal and sinus symptoms are more likely to respond to a questionnaire about these symptoms than individuals without these symptoms. Therefore the prevalence may be overestimated. The prevalence found in this study was slightly higher than reported for the Netherlands on the basis of the Ouderkerk data only (CRS 14.3%) and also confirms the relatively high prevalence of CRS in the Netherlands by comparison with the average in Europe (11%) [20] and the US (12%). [21]

We also realise that some of the subjects reporting allergies tested negative in skin-prick testing and that others were not aware of the allergic basis for their complaints. In an Italian study, 79% of the participants reporting AR had either a positive skin prick test or at least one specific IgE measurement ≥ 0.35 kU/l. [22] Twenty-eight percent of the participants in a Turkish study who answered ‘yes’ to the question ‘Do you have or have you ever had any nasal allergies, including hay fever?’ had a positive skin prick test. [23]

The associations found between AR, ARS, CRS and asthma and eczema concur with other studies evaluating the comorbidities of AR. [24, 25]

We found that Caucasians were less likely to have AR than most other ethnicities. In an English study in general practice, significantly fewer Southern Irish participants and significantly more West Indian women consulted a general practitioner for AR than the native British population. [26] By contrast, Salo et al. found that non-Hispanic whites reported more hay fever than non-Hispanic blacks, Mexican Americans and other ethnicities. [27]

Interestingly, we found that full-time housewives/househusbands were significantly less likely to have AR than respondents with jobs. This is a new finding that could possibly be explained by occupational AR in the latter group. It is known that occupational exposure is related to upper airway disease.[28] Occupational AR may result from a wide variety of high-molecular-weight agents and some low-molecular-weight agents. Examples of occupations at increased risk are furriers, bakers, livestock breeders, food-processing workers, veterinarians, farmers, electronic/electrical products assemblers and boat builders. [29–31] Furthermore, AR has been found be more prevalent in medical professionals than in office workers and in cleaners [32, 33].

Occupational status might reflect socioeconomic status and may be of influence on the prevalence of ARS, CRS and AR. In a recent study by Philpott factors such as occupation, highest academic qualification, rural/urban location, duration of residency, proximity to crops, postcode, annual income, ethnicity, household occupancy and social class were studied in relation to CRS. No significant differences were found after adjusting for age and sex.[34]

Hirsch used the history of receiving Medical Assistance as surrogate for socioeconomic status and found that this was associated with CRS.[21]

Kilty found that participants with an educational level of high school of less report higher sinus symptom scores than participants with post-secondary education. Their Lund MacKay score on CT however is not significantly different.[35] This indicates that socioeconomic factors may be of influence on reporting of (severity) of symptoms. Unfortunately, we do not have information about the socioeconomic status of our participants.

Conflicting results have been found in previous studies about the effect of smoking on AR. [5, 27, 36–39] In our study, we found a negative association between smoking and AR. The healthy smoker phenomenon could explain why our study and some other studies have shown that smokers are less likely to have AR than non-smokers. [5, 36–38] It is possible that allergy subjects are less likely to start smoking and more likely to quit smoking. Smoking may have an immunosuppressive effect and reduce the number of IgE sensitizations. [27, 39]

In our multivariate analysis, we did not find any association between place of residence and AR. However, several studies have found a link between living environment and nasal symptoms/AR. People living close to heavy traffic and in cities reported nasal symptoms more often. [40–42] It is very probable that the wide range of living conditions in the three locations was such that these differences could not be found.

The present survey confirmed the findings in the literature indicating a significant correlation between asthma and CRS and AR, but not between asthma and ARS. [43–46] This could be explained by the fact that CRS and AR are chronic diseases, as is asthma. The finding supports the unified airway theory and the conclusion that ARS and CRS are two different diseases.

The relation of an adverse response to painkillers and CRS (with nasal polyps) is not surprising because they often occur together with asthma as part of AERD (aspirin-exacerbated respiratory disease).[2]Itchy rash as defined in our study might fit the diagnosis of urticaria.

When we look at the relation of urticaria with ARS and CRS in other studies, we found that chronic urticaria are often related to infections (in general) in several studies.[47–50] Positive nasal swabs were more often found in patients with urticaria than in controls.[48]

In this study, Caucasians tended to have a higher prevalence of ARS and a lower prevalence of chronic respiratory conditions as CRS and AR by comparison with other ethnicities. It is difficult to compare these data with previous studies because of differences in the definitions of race/ethnicity and rhinosinusitis (ARS and CRS were not studied separately elsewhere). Our data confirm an earlier study by Tan, in which the local population of Singapore had more CRS than the Caucasian population. The local population of Singapore consisted of Chinese (71,2%), Malay (8,9%), Indian (13,5%) and other ethnicities (6,6%). [51] A survey from the US found associations between the prevalence of rhinosinusitis (defined as a positive response to the question: ‘During the past twelve months, have you had sinusitis or sinus problems?’) and female gender, non-Hispanic white or black race, higher income status and higher educational level. [12] Contrary to our data, Hirsch et al. found that non-whites had a lower risk of meeting the EPOS CRS criteria than whites in the United States (OR 0.53). [21]

We found that women were more likely to have ARS, but not CRS. This concurs with the study by Hirsch. [21] Almost 15% of the respondents from the 2002–2005 National Health Interview Survey of the United States had been diagnosed with rhinosinusitis in the previous year (doctor’s diagnosis, no differentiation between ARS and CRS). This prevalence was lower in the Asian (7%) and Hispanic populations (8.6–8.8%) than in the black population (13.3–14.4%) and the white population (13.0–16.0%). [13, 14] In a retrospective study in children it was found that there were more white children in the CRS group (77%) than in the group without CRS (47%). (CRS group: 77% white, 10% black, 13% other; control group: 47% white, 33% black, 20% other). [52] Different study types with different populations and different definitions of ethnicities and rhinosinusitis may explain the conflicting findings on this subject. It may be a genetical issue, but habits/environment may also play a role. Further research is needed to elucidate the findings regarding ethnicity in our study.We did not find any significant association between CRS and work/occupation. Earlier, Thilsing et al. did find an increased prevalence of CRS in subjects working in a cleaning job. [53] A correlation has also been found between occupational exposure to low- and high-molecular-weight irritants and the number of FESS (functional endoscopic sinus surgery) procedures in patients with CRS. [54] However, a recent review by Sundaresan evaluating 41 articles that discussed occupational and environmental influences on CRS stated that the literature at present allows us to draw very few conclusions about the role of hazardous occupational or environmental exposures in CRS, leaving a critical knowledge gap regarding potentially modifiable risk factors for disease onset and progression. [55] More research is definitely needed to elucidate the effect of occupational exposure on CRS.

We found a positive link between smoking and CRS and ARS, confirming other studies. [3, 12, 21, 53, 54]

In conclusion, this study found new associations between different upper airway diseases and relevant factors. It is again clear that chronic upper airway diseases like AR and CRS are associated with other factors than acute diseases like ARS.

More studies are required evaluating sensitisation and other objective signs of disease to further unravel these observations.

## Supporting information

S1 Appendix (PDF)

S1 Dataset (SAV)

## Abbreviations

AERD: aspirin-exacerbated respiratory disease
ANOVA: analysis of variance
AR: allergic rhinitis
ARS: acute rhinosinusitis
CRS: chronic rhinosinusitis
CI: confidence interval
CT: computed tomography
EPOS: European position paper on rhinosinusitis and nasal polyps
FESS: functional endoscopic sinus surgery
GA^2^LEN: The Global Allergy and Asthma European Network
OR: odds ratio
SPSS: Statistical Package for the Social Sciences

## References

[1] Bauchau V, Durham SR. Prevalence and rate of diagnosis of allergic rhinitis in Europe. The European respiratory journal. 2004;24(5):758–64. Epub 2004/11/02. doi: 10.1183/09031936.04.00013904 . 15516669 10.1183/09031936.04.00013904

[2] Fokkens WJ, Lund VJ, Mullol J, Bachert C, Alobid I, Baroody F, et al European Position Paper on Rhinosinusitis and Nasal Polyps 2012. Rhinol Suppl. 2012;(23):3–298. 22764607

[3] Hastan D, Fokkens WJ, Bachert C, Newson RB, Bislimovska J, Bockelbrink A, et al Chronic rhinosinusitis in Europe—an underestimated disease. A GA(2)LEN study. Allergy. 2011;66(9):1216–23. doi: 10.1111/j.1398-9995.2011.02646.x 21605125 10.1111/j.1398-9995.2011.02646.x

[4] Katelaris CH, Lee BW, Potter PC, Maspero JF, Cingi C, Lopatin A, et al Prevalence and diversity of allergic rhinitis in regions of the world beyond Europe and North America. Clin Exp Allergy. 2012;42(2):186–207. Epub 2011/11/19. doi: 10.1111/j.1365-2222.2011.03891.x . 22092947 10.1111/j.1365-2222.2011.03891.x

[5] Bousquet J, Khaltaev N, Cruz AA, Denburg J, Fokkens WJ, Togias A, et al Allergic Rhinitis and its Impact on Asthma (ARIA) 2008 update (in collaboration with the World Health Organization, GA(2)LEN and AllerGen). Allergy. 2008;63 Suppl 86:8–160. doi: 10.1111/j.1398-9995.2007.01620.x.:8–160 18331513 10.1111/j.1398-9995.2007.01620.x

[6] Bousquet J, Lund VJ, van CP, Bremard-Oury C, Mounedji N, Stevens MT, et al Implementation of guidelines for seasonal allergic rhinitis: a randomized controlled trial. Allergy. 2003;(8):41. 10.1034/j.1398-9995.2003.00076.x 12859551

[7] Baroody FM, Mucha SM, deTineo M, Naclerio RM. Evidence of maxillary sinus inflammation in seasonal allergic rhinitis. Otolaryngol Head Neck Surg. 2012;146(6):880–6. doi: 10.1177/0194599811435972 22301108 10.1177/0194599811435972

[8] Fokkens WJ, Lund VJ, Mullol J, Bachert C, Alobid I, Baroody F, et al European Position Paper on Rhinosinusitis and Nasal Polyps 2012. Rhinology Supplement. 2012;(23):3 p preceding table of contents, 1–298. Epub 2012/07/07. . 22764607

[9] Krouse JH, Brown RW, Fineman SM, Han JK, Heller AJ, Joe S, et al Asthma and the unified airway. Otolaryngol Head Neck Surg. 2007;136(5 Suppl):S75–106. 17462497 10.1016/j.otohns.2007.02.019

[10] Feng CH, Miller MD, Simon RA. The united allergic airway: connections between allergic rhinitis, asthma, and chronic sinusitis. American journal of rhinology & allergy. 2012;26(3):187–90. Epub 2012/05/31. doi: 10.2500/ajra.2012.26.3762 ; PubMed Central PMCID: PMCPmc3906509. 22643942 10.2500/ajra.2012.26.3762 PMC3906509

[11] Giavina-Bianchi P, Aun MV, Takejima P, Kalil J, Agondi RC. United airway disease: current perspectives. Journal of asthma and allergy. 2016;9:93–100. Epub 2016/06/04. doi: 10.2147/JAA.S81541 ; PubMed Central PMCID: PMCPmc4872272. 27257389 10.2147/JAA.S81541 PMC4872272

[12] Lieu JE, Feinstein AR. Confirmations and surprises in the association of tobacco use with sinusitis. Arch Otolaryngol Head Neck Surg. 2000;126(8):940–6. 10922224 10.1001/archotol.126.8.940

[13] Nachman KE, Parker JD. Exposures to fine particulate air pollution and respiratory outcomes in adults using two national datasets: a cross-sectional study. Environ Health. 2012;11:25 doi: 10.1186/1476-069X-11-25 22490087 10.1186/1476-069X-11-25 PMC3361500

[14] Soler ZM, Mace JC, Litvack JR, Smith TL. Chronic rhinosinusitis, race, and ethnicity. Am J Rhinol Allergy. 2012;26(2):110–6. doi: 10.2500/ajra.2012.26.3741 22487286 10.2500/ajra.2012.26.3741 PMC3345896

[15] Hoffmans R, Schermer T, van der Linde K, Bor H, van Boven K, van Weel C, et al Rhinosinusitis in morbidity registrations in Dutch General Practice: a retro-spective case-control study. BMC family practice. 2015;16:120 Epub 2015/09/13. doi: 10.1186/s12875-015-0332-8 ; PubMed Central PMCID: PMCPMC4567828. 26362443 10.1186/s12875-015-0332-8 PMC4567828

[16] Hoffmans R, Schermer T, van WC, Fokkens W. Management of rhinosinusitis in Dutch general practice. Prim Care Respir J. 2011;20(1):64–70. doi: 10.4104/pcrj.2010.00064 21311844 10.4104/pcrj.2010.00064 PMC6549798

[17] Tan BK, Kern RC, Schleimer RP, Schwartz BS. Chronic rhinosinusitis: the unrecognized epidemic. American journal of respiratory and critical care medicine. 2013;188(11):1275–7. Epub 2013/12/03. doi: 10.1164/rccm.201308-1500ED ; PubMed Central PMCID: PMCPMC3919079. 24289768 10.1164/rccm.201308-1500ED PMC3919079

[18] Tomassen P, Newson RB, Hoffmans R, Lotvall J, Cardell LO, Gunnbjornsdottir M, et al Reliability of EP3OS symptom criteria and nasal endoscopy in the assessment of chronic rhinosinusitis—a GA(2) LEN study. Allergy. 2011;66(4):556–61. doi: 10.1111/j.1398-9995.2010.02503.x 21083566 10.1111/j.1398-9995.2010.02503.x

[19] Park DY, Lee EJ, Kim JH, Kim YS, Jung CM, Kim KS. Correlation between symptoms and objective findings may improve the symptom-based diagnosis of chronic rhinosinusitis for primary care and epidemiological studies. BMJ open. 2015;5(12):e009541 Epub 2015/12/18. doi: 10.1136/bmjopen-2015-009541 ; PubMed Central PMCID: PMCPmc4691778. 26674502 10.1136/bmjopen-2015-009541 PMC4691778

[20] Hastan D, Fokkens WJ, Bachert C, Newson RB, Bislimovska J, Bockelbrink A, et al Chronic rhinosinusitis in Europe—an underestimated disease. A GA(2)LEN study. Allergy. 2011;66(9):1216–23. Epub 2011/05/25. doi: 10.1111/j.1398-9995.2011.02646.x . 21605125 10.1111/j.1398-9995.2011.02646.x

[21] Hirsch AG, Stewart WF, Sundaresan AS, Young AJ, Kennedy TL, Scott Greene J, et al Nasal and sinus symptoms and chronic rhinosinusitis in a population-based sample. Allergy. 2017;72(2):274–81. Epub 2016/09/04. doi: 10.1111/all.13042 . 27590749 10.1111/all.13042 PMC5497579

[22] Olivieri M, Verlato G, Corsico A, Lo Cascio V, Bugiani M, Marinoni A, et al Prevalence and features of allergic rhinitis in Italy. Allergy. 2002;57(7):600–6. Epub 2002/07/09. . 12100300 10.1034/j.1398-9995.2002.23537.x

[23] Ozdemir N, Ucgun I, Metintas S, Kolsuz M, Metintas M. The prevalence of asthma and allergy among university freshmen in Eskisehir, Turkey. Respir Med. 2000;94(6):536–41. doi: 10.1053/rmed.1999.0728 10921756 10.1053/rmed.1999.0728

[24] Bousquet J, Schunemann HJ, Samolinski B, Demoly P, Baena-Cagnani CE, Bachert C, et al Allergic Rhinitis and its Impact on Asthma (ARIA): achievements in 10 years and future needs. J Allergy Clin Immunol. 2012;130(5):1049–62. Epub 2012/10/09. doi: 10.1016/j.jaci.2012.07.053 . 23040884 10.1016/j.jaci.2012.07.053

[25] Seidman MD, Gurgel RK, Lin SY, Schwartz SR, Baroody FM, Bonner JR, et al Clinical practice guideline: allergic rhinitis executive summary. Otolaryngol Head Neck Surg. 2015;152(2):197–206. doi: 10.1177/0194599814562166 25645524 10.1177/0194599814562166

[26] Gillam SJ, Jarman B, White P, Law R. Ethnic differences in consultation rates in urban general practice. BMJ. 1989;299(6705):953–7. 2508951 10.1136/bmj.299.6705.953 PMC1837829

[27] Salo PM, Calatroni A, Gergen PJ, Hoppin JA, Sever ML, Jaramillo R, et al Allergy-related outcomes in relation to serum IgE: results from the National Health and Nutrition Examination Survey 2005–2006. J Allergy Clin Immunol. 2011;127(5):1226–35.e7. Epub 2011/02/16. doi: 10.1016/j.jaci.2010.12.1106 ; PubMed Central PMCID: PMCPmc3108140. 21320720 10.1016/j.jaci.2010.12.1106 PMC3108140

[28] Hox V, Steelant B, Fokkens W, Nemery B, Hellings PW. Occupational upper airway disease: how work affects the nose. Allergy. 2014;69(3):282–91. Epub 2014/01/09. doi: 10.1111/all.12347 . 24397491 10.1111/all.12347

[29] Moscato G, Vandenplas O, Van Wijk RG, Malo JL, Perfetti L, Quirce S, et al EAACI position paper on occupational rhinitis. Respiratory research. 2009;10:16 Epub 2009/03/05. doi: 10.1186/1465-9921-10-16 ; PubMed Central PMCID: PMCPmc2654869. 19257881 10.1186/1465-9921-10-16 PMC2654869

[30] Hytonen M, Kanerva L, Malmberg H, Martikainen R, Mutanen P, Toikkanen J. The risk of occupational rhinitis. International archives of occupational and environmental health. 1997;69(6):487–90. Epub 1997/01/01. . 9215937 10.1007/s004200050178

[31] Stevens WW, Grammer LC, 3rd. Occupational rhinitis: an update. Current allergy and asthma reports. 2015;15(1):487 Epub 2014/11/29. doi: 10.1007/s11882-014-0487-8 . 25430949 10.1007/s11882-014-0487-8

[32] Radon K, Gerhardinger U, Schulze A, Zock JP, Norback D, Toren K, et al Occupation and adult onset of rhinitis in the general population. Occup Environ Med. 2008;65(1):38–43. doi: 10.1136/oem.2006.031542 17664253 10.1136/oem.2006.031542

[33] Moscato G, Siracusa A. Rhinitis guidelines and implications for occupational rhinitis. Curr Opin Allergy Clin Immunol. 2009;9(2):110–5. 19326505 10.1097/aci.0b013e328328cfe7

[34] Philpott C, Erskine S, Hopkins C, Coombes E, Kara N, Sunkareneni V, et al A case-control study of medical, psychological and socio-economic factors influencing the severity of chronic rhinosinusitis. Rhinology. 2016;54(2):134–40. Epub 2016/05/14. doi: 10.4193/Rhin15.272 . 27172454 10.4193/Rhino15.272

[35] Kilty SJ, McDonald JT, Johnson S, Al-Mutairi D. Socioeconomic status: a disease modifier of chronic rhinosinusitis? Rhinology. 2011;49(5):533–7. Epub 2011/11/30. doi: 10.4193/Rhino10.298 . 22125783 10.4193/Rhino10.298

[36] Eriksson J, Ekerljung L, Sundblad BM, Lotvall J, Toren K, Ronmark E, et al Cigarette smoking is associated with high prevalence of chronic rhinitis and low prevalence of allergic rhinitis in men. Allergy. 2013;68(3):347–54. doi: 10.1111/all.12095 23346908 10.1111/all.12095

[37] Higgins TS, Reh DD. Environmental pollutants and allergic rhinitis. Curr Opin Otolaryngol Head Neck Surg. 2012;20(3):209–14. doi: 10.1097/MOO.0b013e3283534821 22487789 10.1097/MOO.0b013e3283534821

[38] Saulyte J, Regueira C, Montes-Martinez A, Khudyakov P, Takkouche B. Active or passive exposure to tobacco smoking and allergic rhinitis, allergic dermatitis, and food allergy in adults and children: a systematic review and meta-analysis. PLoS Med. 2014;11(3):e1001611 doi: 10.1371/journal.pmed.1001611 24618794 10.1371/journal.pmed.1001611 PMC3949681

[39] Olivieri M, Heinrich J, Schlunssen V, Anto JM, Forsberg B, Janson C, et al The risk of respiratory symptoms on allergen exposure increases with increasing specific IgE levels. Allergy. 2016;71:859–68. Epub 2016/01/15. doi: 10.1111/all.12841 . 26764559 10.1111/all.12841

[40] Montnemery P, Popovic M, Andersson M, Greiff L, Nyberg P, Lofdahl CG, et al Influence of heavy traffic, city dwelling and socio-economic status on nasal symptoms assessed in a postal population survey. Respir Med. 2003;97(8):970–7. 12924526 10.1016/s0954-6111(03)00126-4

[41] Jang AS, Jun YJ, Park MK. Effects of air pollutants on upper airway disease. Curr Opin Allergy Clin Immunol. 2016;16(1):13–7. doi: 10.1097/ACI.0000000000000235 26658014 10.1097/ACI.0000000000000235

[42] Lindgren A, Stroh E, Nihlen U, Montnemery P, Axmon A, Jakobsson K. Traffic exposure associated with allergic asthma and allergic rhinitis in adults. A cross-sectional study in southern Sweden. Int J Health Geogr. 2009;8:25 doi: 10.1186/1476-072X-8-25 19419561 10.1186/1476-072X-8-25 PMC2687434

[43] Chung SD, Chen PY, Lin HC, Hung SH. Comorbidity profile of chronic rhinosinusitis: a population-based study. Laryngoscope. 2014;124(7):1536–41. doi: 10.1002/lary.24581 24395611 10.1002/lary.24581

[44] Dixon AE. Rhinosinusitis and asthma: the missing link. Curr Opin Pulm Med. 2009;15(1):19–24. doi: 10.1097/MCP.0b013e32831da87e 19077701 10.1097/MCP.0b013e32831da87e PMC2774711

[45] Hellings PW, Fokkens WJ. Allergic rhinitis and its impact on otorhinolaryngology. Allergy. 2006;61(6):656–64. doi: 10.1111/j.1398-9995.2006.01109.x 16677233 10.1111/j.1398-9995.2006.01109.x

[46] Hirsch AG, Yan XS, Sundaresan AS, Tan BK, Schleimer RP, Kern RC, et al Five-year risk of incident disease following a diagnosis of chronic rhinosinusitis. Allergy. 2015;70(12):1613–21. Epub 2015/09/04. doi: 10.1111/all.12759 ; PubMed Central PMCID: PMCPmc4715505. 26332371 10.1111/all.12759 PMC4715505

[47] Buss YA, Garrelfs UC, Sticherling M. Chronic urticaria—which clinical parameters are pathogenetically relevant? A retrospective investigation of 339 patients. Journal der Deutschen Dermatologischen Gesellschaft = Journal of the German Society of Dermatology: JDDG. 2007;5(1):22–9. Epub 2007/01/19. doi: 10.1111/j.1610-0387.2007.06194.x . 17229201 10.1111/j.1610-0387.2007.06194.x

[48] Ertam I, Biyikli SE, Yazkan FA, Aytimur D, Alper S. The frequency of nasal carriage in chronic urticaria patients. Journal of the European Academy of Dermatology and Venereology: JEADV. 2007;21(6):777–80. Epub 2007/06/15. doi: 10.1111/j.1468-3083.2006.02083.x . 17567307 10.1111/j.1468-3083.2006.02083.x

[49] Wedi B, Raap U, Wieczorek D, Kapp A. Urticaria and infections. Allergy, asthma, and clinical immunology: official journal of the Canadian Society of Allergy and Clinical Immunology. 2009;5(1):10 Epub 2010/01/13. doi: 10.1186/1710-1492-5-10 ; PubMed Central PMCID: PMCPMC2804274. 20066173 10.1186/1710-1492-5-10 PMC2804274

[50] Olze H, Zuberbier T. Comorbidities between nose and skin allergy. Current opinion in allergy and clinical immunology. 2011;11(5):457–63. Epub 2011/08/09. doi: 10.1097/ACI.0b013e32834a9764 . 21822129 10.1097/ACI.0b013e32834a9764

[51] Tan TY, Lin M, Cheah FK, Koh DM. Distribution patterns of inflammatory sinonasal diseases. Singapore Med J. 1998;39(2):59–63. 9652178

[52] Smith DF, Ishman SL, Tunkel DE, Boss EF. Chronic rhinosinusitis in children: race and socioeconomic status. Otolaryngol Head Neck Surg. 2013;149(4):639–44. doi: 10.1177/0194599813498206 23884283 10.1177/0194599813498206

[53] Thilsing T, Rasmussen J, Lange B, Kjeldsen AD, Al-Kalemji A, Baelum J. Chronic rhinosinusitis and occupational risk factors among 20- to 75-year-old Danes-A GA(2) LEN-based study. Am J Ind Med. 2012;55(11):1037–43. doi: 10.1002/ajim.22074 22648974 10.1002/ajim.22074

[54] Hox V, Delrue S, Scheers H, Adams E, Keirsbilck S, Jorissen M, et al Negative impact of occupational exposure on surgical outcome in patients with rhinosinusitis. Allergy. 2012;67(4):560–5. doi: 10.1111/j.1398-9995.2011.02779.x 22229752 10.1111/j.1398-9995.2011.02779.x

[55] Sundaresan AS, Hirsch AG, Storm M, Tan BK, Kennedy TL, Greene JS, et al Occupational and environmental risk factors for chronic rhinosinusitis: a systematic review. International forum of allergy & rhinology. 2015;5(11):996–1003. Epub 2015/06/17. doi: 10.1002/alr.21573 ; PubMed Central PMCID: PMCPmc4681694. 26077513 10.1002/alr.21573 PMC4681694

