# Supplementary material for: Acute and chronic rhinosinusitis and allergic rhinitis in relation to comorbidity, ethnicity and environment
Source: PLoS One. 2018 Feb 5;13(2):e0192330. doi: 10.1371/journal.pone.0192330 (PMC5798836; doi:10.1371/journal.pone.0192330)
Supplement: S1 Appendix — (PDF) [file pone.0192330.s001.pdf]

GA2LEN SURVEY QUESTIONNAIRE

TIP

TO ANSWER THE QUESTIONS PLEASE TICK THE APPROPRIATE BOX — **NO** ☐ **YES** ☑ √

IF YOU ARE UNSURE OF THE ANSWER PLEASE CHOOSE 'NO'

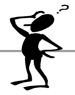

1. Have you had wheezing or whistling in your chest at any time **in the last 12 months**? — NO ☐ YES ☐

IF 'NO' GO TO QUESTION 2 — ***IF 'YES' GO TO QUESTION 1.1***

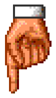

1.1 Have you been at all breathless when the wheezing noise was present? — NO ☐ YES ☐

1.2 Have you had this wheezing or whistling when you did not have a cold? — NO ☐ YES ☐

2. Have you woken up with a feeling of tightness in your chest at any time **in the last 12 months**? — NO ☐ YES ☐

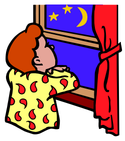

3. Have you been woken by an attack of shortness of breath at any time **in the last 12 months**? — NO ☐ YES ☐

4. Have you been woken by an attack of coughing at any time **in the last 12 months?** — NO ☐ YES ☐

5. Do you bring up phlegm from your chest on most days for as much as three months each year? — NO ☐ YES ☐

6. Have you **ever** had asthma? — NO ☐ YES ☐

IF 'NO' GO TO QUESTION 7 — ***IF 'YES' GO TO QUESTION 6.1***

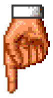

6.1 How old were you when you had your first attack of asthma? (If unsure, give your best guess!) — YEARS ☐☐

6.2 Have you **ever** been hospitalised with asthma? — NO ☐ YES ☐

6.3 Have you had an attack of asthma **in the last 12 months?** — NO ☐ YES ☐

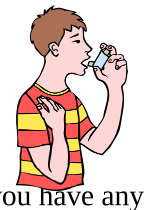

6.4 Are you **currently** taking any medicine (including inhalers, aerosols or tablets) for **asthma?** — NO ☐ YES ☐

7. Do you have any nasal allergies including hay fever? — NO ☐ YES ☐

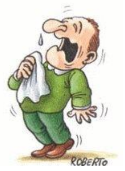

«id»

*IF '**NO**' GO TO QUESTION 8* | *IF 'YES' GO TO QUESTION 7.1*

7.1 Have you been troubled by nasal allergies **in the last 12 months?** NO ☐ YES ☐

7.2 Have you ever been troubled by nasal allergies for **more than 4 days in any one week**? NO ☐ YES ☐

7.3 If yes did this happen for **more than 4 weeks continuously?** NO ☐ YES ☐

8. Has your nose been blocked **for more than 12 weeks during the last 12 months?** NO ☐ YES ☐

9. Have you had pain or pressure around the forehead, nose or eyes **for more than 12 weeks during the last 12 months?** NO ☐ YES ☐

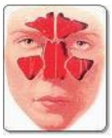

10. Have you had discoloured nasal discharge (snot) or discoloured mucus in the throat **for more than 12 weeks during the last 12 months?** NO ☐ YES ☐

11. Has your sense of smell been reduced or absent **for more than 12 weeks during the last 12 months?** NO ☐ YES ☐

12. Has a doctor **ever** told you that you have **chronic** sinusitis? NO ☐ YES ☐

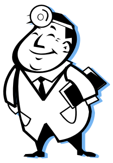

12. A In the past 12 months, have you had at least one episode of at least ten days where you had a blocked nose, discoloured nasal discharge (snot) **and** pain or pressure over the sinuses? NO ☐ YES ☐

*IF '**NO**' GO TO QUESTION 13* | *IF 'YES'GO TO QUESTION 12.A.1.*

12.A.1. How many of these episodes of at least 10 days where you had a blocked nose, discoloured nasal discharge (snot) **and** pain or pressure over the sinuses did you have **in the past 12 months**?

1 2 3 4 >4

12.A.2. Have you visited a doctor for one of these episodes? NO ☐ YES ☐

12.A.3. Have you received antibiotics for one of these episodes? NO ☐ YES ☐

12.A.4. Have you received a corticosteroid nose spray for one of these episodes? NO ☐ YES ☐

13. Have you ever had an itchy rash that was coming and going **for at least 6 months?** NO ☐ YES ☐

*IF '**NO**', GO TO QUESTION 1* | *IF 'YES' GO TO QUESTION 13.1*

«id»

NO YES

13.1 Have you had this itchy rash **<u>in the last 12 months</u>**? ☐ ☐

NO YES

13.2 Does this affect **<u>only</u>** your hands? ☐ ☐

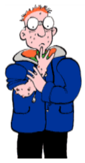

NO YES

14. Have you ever had eczema or any kind of skin allergy? ☐ ☐

NO YES

15. Have you ever had any difficulty with your breathing within 3 hours after taking a pain killer? ☐ ☐

IF 'NO' GO TO QUESTION 16 ***IF 'YES' GO TO QUESTION 15.1***

15.1 Please write the name of the tablet? ……………………………………………………………

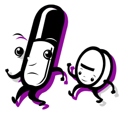

NO YES

16. Have you ever smoked for as long as a year? ☐ ☐

['YES' means at least one cigarette per day or one cigar per week for one year]

IF 'NO' GO TO QUESTION 17 ***IF 'YES' GO TO QUESTION 16.1***

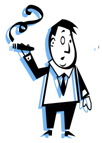

YEARS

16.1 How old were you when you started smoking? ☐☐

NO YES

16.2 Have you smoked at all in the **last month**? ☐ ☐

IF 'YES' GO TO QUESTION 16.3 ***IF 'NO' GO TO 16.2.1***

YEARS

16.2.1 How old were you when you stopped smoking? ☐☐

Cigarettes per day

16.3 **<u>On average</u>** how much do you (or did you) smoke? ☐☐

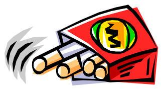

17. Are you currently: ***Tick one box only!***

**a.** employed	☐	1.
**b.** self-employed	☐	2.
**c.** unemployed	☐	3.
**d.** not working because of poor health	☐	4.
**e.** full-time house person	☐	5.
**f.** full-time student	☐	6.
**g.** retired	☐	7.
**h.** other	☐	8.

18. Are you currently working: NO YES

a. As a health care worker (e.g. as a nurse, medical technician, ☐ ☐

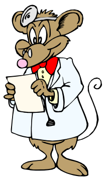

doctor, paramedic or similar)?

NO YES

b. In a job that is mainly involved with any sort of cleaning? ☐ ☐

NO YES

19.1 Do you understand the language in which this questionnaire is composed? ☐ ☐

19.2 Which language do you speak most when you're at home? ________

19.3 Which language do you speak most when you're away from home? ________

20.1 In which country were you born? ________

20.2 In which country was your father born? ________

20.3 In which country was your mother born? ________

Tick one box only!

20.4 What is your ethnicity?

- **a.** Caucasian/white ☐ 1.
- **b.** Asian ☐ 2.
- **c.** African/Creole ☐ 3.
- **d.** Latin-American ☐ 4.
- **e.** Hindustani ☐ 5.
- **f.** Mediterranean ☐ 6.
- **g.** Other (please specify): ________

YEARS

20.5 How many years have you been living in The Netherlands? ________

DAY MONTH YEAR

21. What is your date of birth? ☐☐ ☐☐ **19**☐☐

DAY MONTH YEAR

22. What is today's date? ☐☐ ☐☐ **20**☐☐

MALE FEMALE

23. Are you male or female? ☐ ☐

24. What is your postal code? ☐☐☐☐ ☐☐

NO YES

May we contact you again for further scientific research? ☐ ☐

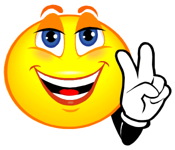

Thanks for your help!

«id»
